# Retroperitoneal lymphangioma as the final diagnosis of a middle-aged woman with abdominal pain: a case report

**DOI:** 10.1186/s13256-023-03803-6

**Published:** 2023-03-15

**Authors:** Hamid Asadzadeh Aghdaei, Amirhassan Rabbani, Amir Sadeghi, Hamid Rezvani, Ghazal Sherkat, Naghmeh Salarieh, Pardis Ketabi Moghadam

**Affiliations:** 1 Research Institute for Gastroenterology and Liver Diseases, Shahid Beheshti University of Medical Sciences, Tehran, Iran; 2 Surgery Department of Taleghani Hospital, Shahid Beheshti University of Medical Sciences, Tehran, Iran; 3 Division of Medical Oncology, Taleghani Hospital Cancer Center, Shahid Beheshti University of Medical Sciences, Tehran, Iran; 4 Medicine Faculty of Mashhad Branch, Islamic Azad University, Mashhad, Iran

**Keywords:** Retroperitoneal lymphangioma, Pancreatic ductal adenocarcinoma, Pancreatic cystic neoplasm

## Abstract

**Background:**

Lymphangiomas are lesions attributed to congenital malformations of the lymphatic system, or acquired chronic obstruction of the lymphatic network due to trauma, radiation, surgical manipulation, inflammation, or infection. Overall, lymaphangiomas are rare, and particularly, retroperitoneal lymphangiomas are far more uncommon per reported cases.

**Case presentation:**

A 49-year-old Iranian woman presented with a progressive abdominal pain since approximately 1 month before admission. She was found to have a retroperitoneal lymphangioma after a precise radiological and surgical workup.

**Conclusion:**

Retroperitoneal lymphangiomas are rare lesions, sometimes indistinguishable from malignant lesions originating from pancreas and adjacent organs. Complete surgical removal and histologic evaluation of the lesion is the gold standard of treatment and diagnosis.

## Introduction

Lymphangiomas are rare benign lesions originating from lymphatic system [1]. They can be detected at any age, but the infantile type is more common. Any part of the body can be involved, although head and neck, and axilla are more commonly affected. Abdominal lymphangiomas, especially retroperitoneal forms, are found to be rare. It is estimated that less than 1% of overall detected lymphangiomas are located in retroperitoneum [2, 3]. Their clinical presentation is entirely dependent on the place they have arisen from. The compressive effect of the tumor on the adjacent organs determines the symptoms. Of note, retroperitoneal forms are usually manifested by abdominal pain and palpable masses [4]. Ultrasound (US), computed tomography (CT), and magnetic resonance imaging (MRI) are modalities used for evaluation of these tumors, which clearly manifest their cystic nature. However, the diagnostic accuracy of imaging is not sufficient to differentiate these lesions from other cystic/solid-cystic lesions. This may be in part due to their inflammatory changes which obscures their lymphatic origin. Given that, histologic confirmation is a necessity for definite diagnosis of lymphangiomas [5, 6]. Complete resection of these lesions is recognized as the treatment of choice [6].

## Case presentation

**Fig. 1:**
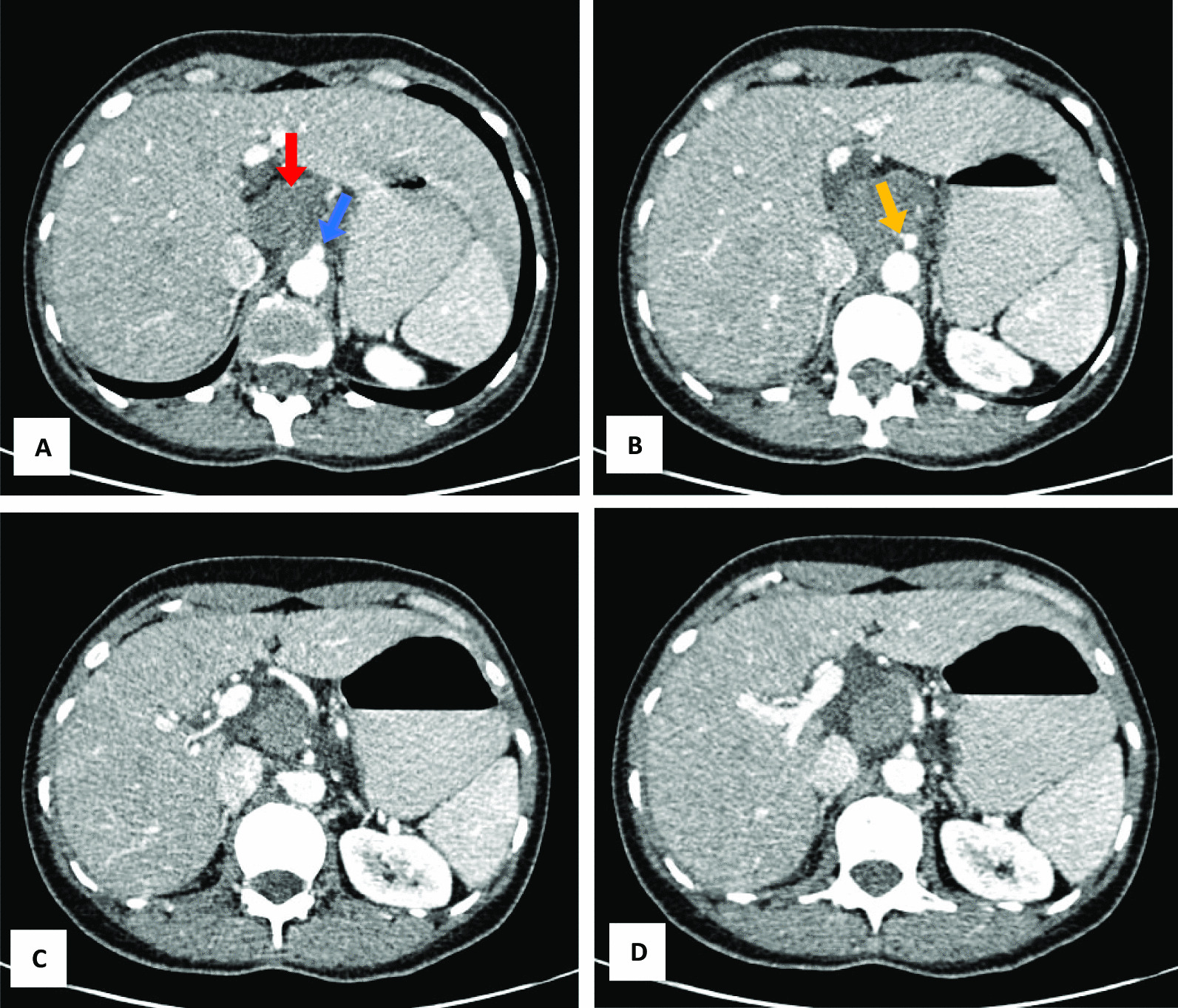
**A**–**D** Abdominopelvic CT scan reveals a well-defined hypodense lesion (**A** marked with red arrow) measuring about 45 × 26 mm^2^ in size, which resembles a soft-tissue mass of unknown origin, without clear enhancement near celiac trunk (**A** marked with blue arrow), and abutting left gastric artery (**B** its origin is marked with yellow arrow)

**Fig. 2:**
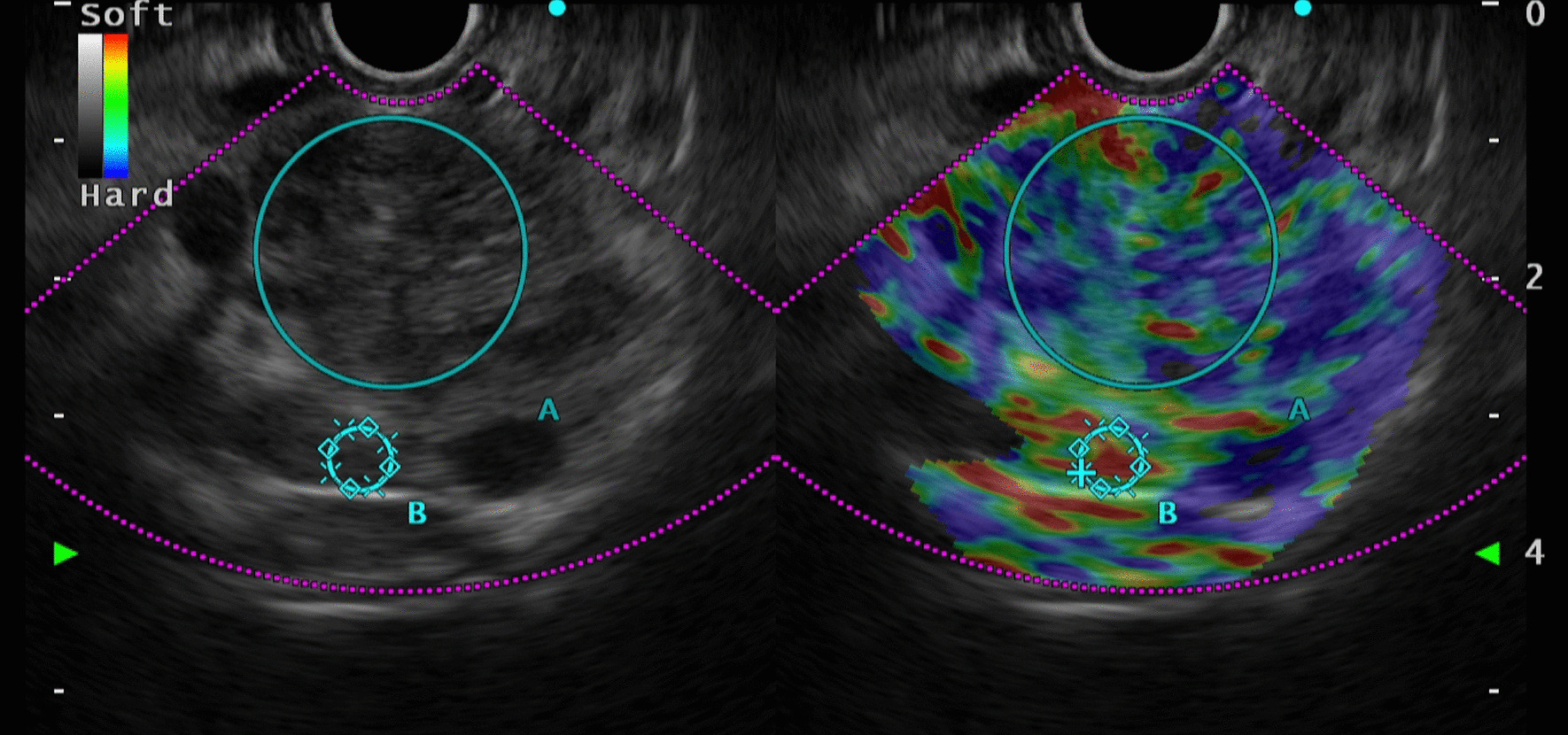
Endoscopic ultrasound study demonstrated a hypoechoic lesion measuring 43 × 35 mm^2^ lying at the posterior wall of the stomach. Tumor encasement of the left gastric artery and a short segment of celiac trunk without obstruction was detected. Elastography strain ratio of the lesion was estimated to be 22

**Fig. 3:**
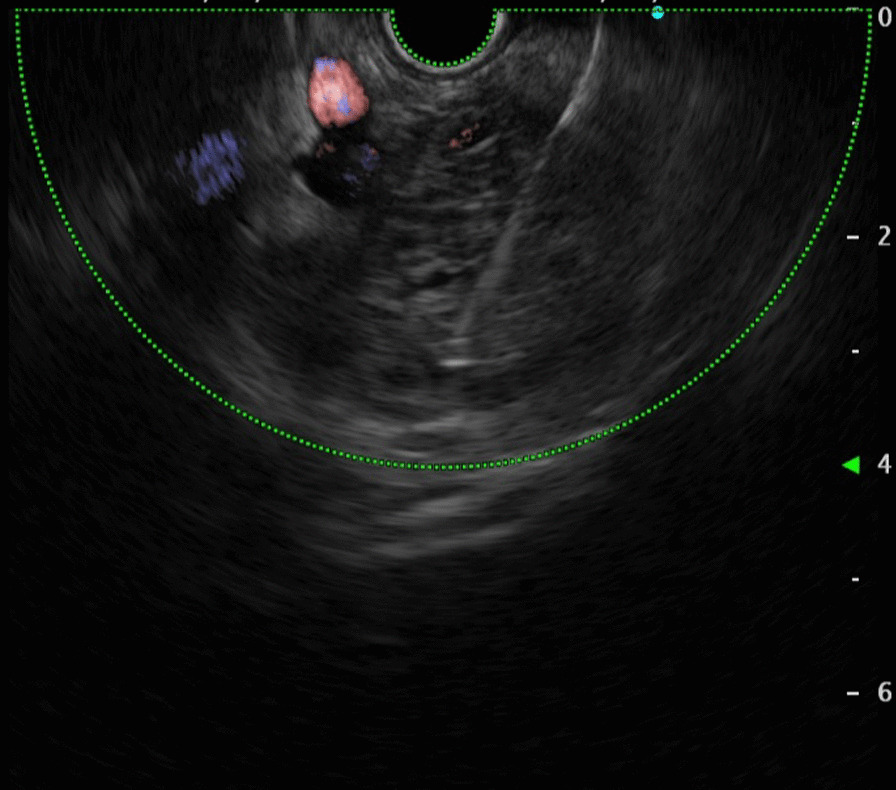
Fine needle aspiration of the lesion

**Fig. 4:**
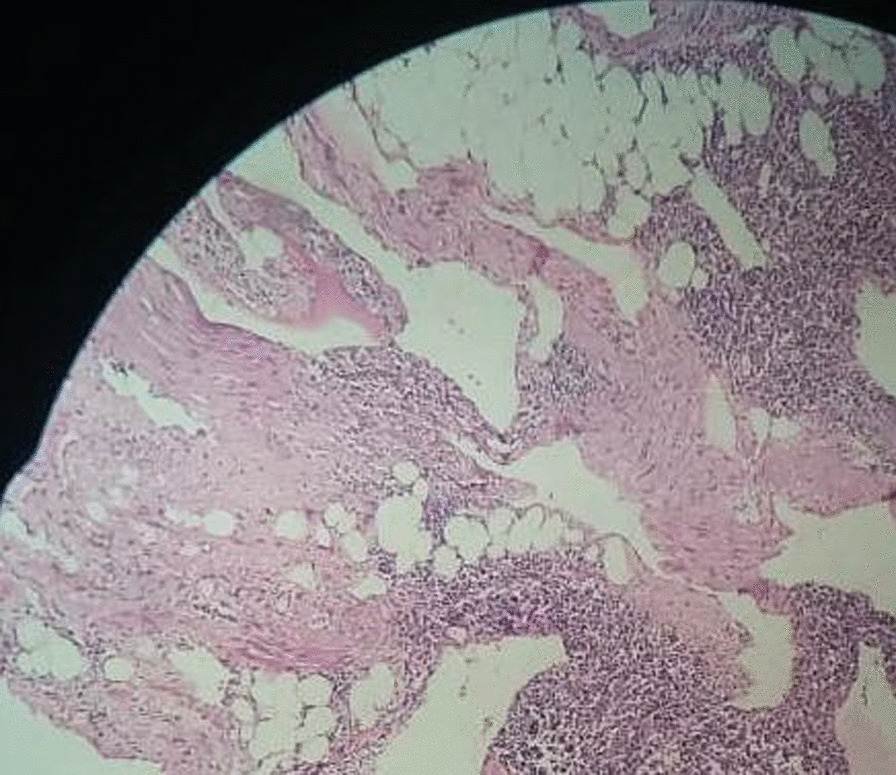
Large lymphatic channels in loose connective tissue stroma with peripheral lymphoid aggregations compatible with lymphangioma

A 49-year-old woman presented to the gastroenterology clinic of Taleghani Hospital, a tertiary academic hospital with a persistent epigastric pain radiating to the back for 1 month before admission. The nature of the abdominal pain was exacerbating over time. It did not alter by fasting or eating. She denied any alteration in bowel habits, but recently suffered from poor appetite, early satiety, and nausea after eating. She did not have any systemic signs and symptoms. Her past medical history was unremarkable except for uterine fibroids. Her family history was positive for pancreatic ductal adenocarcinoma and gastric adenocarcinoma in her second-degree-relatives. On admission, she was uncomfortable but her vital signs were within normal limits. Her physical examination revealed only a mild to moderate tenderness in epigastrium, without any rebound tenderness or guarding. A complete blood count revealed mild anemia with white blood cells (WBC) 5100 cells/mm^3^, hemoglobin (Hb) 11.2 g/dl, and platelets (plt) 210,000/µl. Further evaluations for anemia demonstrated a microcytic and hypochromic anemia with a serum iron profile compatible with iron-deficient anemia. Erythrocyte sedimentation rate (ESR), renal function tests, liver function tests, pancreatic enzymes, coagulation tests, and serum bilirubin level were all within normal limits. Tumor markers such as cancer antigen 19-9 (CA19-9), α-fetoprotein, and carcinoembryonic antigen (CEA) were all within normal ranges. Abdominopelvic ultrasound revealed a heterogeneous hypo- and hyperechoic lesion about 48 × 40 mm^2^ at the head of the pancreas, adjacent to the liver hilum. Color Doppler study of major abdominal vessels was unremarkable. Spiral chest CT scan was normal. Abdominopelvic CT scan showed a well-defined hypodense lesion measuring about 45 × 46 mm^2^ in size, which resembled a soft-tissue mass of unknown origin, without clear enhancement, near celiac trunk with abutment of left gastric artery. Adjacent mesenteric vessels were severely engorged. An increased gastric wall thickness was noted and porta hepatis lymph nodes (smaller than 10 mm) were also detected (Fig. 1). To precisely assess the mentioned lesion, an endoscopic ultrasound (EUS) was utilized, which demonstrated a hypoechoic lesion measuring about 43 × 45 mm^2^ lying at the posterior wall of the stomach. Abutment of the left gastric artery, and a short segment of celiac trunk without clear obstruction by the lesion was detected. Abutment of celiac branches made us worried about the malignant and progressive potential of the lesion, although normal size of pancreatic duct (PD) and common bile duct (CBD) was against the diagnosis of adenocarcinoma. Elastography strain ratio of the lesion was estimated to be 22 (Fig. 2). To have a definite diagnosis, fine needle aspiration (FNA) using EUS-FNA needle 19-gauge was performed, which was unfortunately indeterminate for malignant cells or other diagnoses (Fig. 3). Inconclusive results of FNA made us proceed with surgical resection of the lesion. Surgical exploration revealed a retroperitoneal tumor adherent to celiac trunk, pancreas, and left gastric artery. It was resected and was sent for pathologic evaluation. Damaged arteries were reconstructed. Histologic assessment of the resected mass revealed large lymphatic channels with peripheral lymphoid aggregations embedded in a loose connective tissue stroma, which are diagnostic for benign vascular neoplasms, including lymphangioma (Fig. 4). The patient hereby signed a written informed consent for participation in this report, as she was explained that her name and data will completely be omitted from the documents and images.

## Discussion

Lymphangiomas are slow-progressing tumors that are not reported to harbor malignant potential [1]. The known etiologies of lymphangioma are the anomalous connection between lymphatic and venous network, which is commonly seen in children, as well as traumatic degeneration of lymphatic system by radiation, inflammation, infection, and surgical resection, resulting in chronic obstruction of lymphatic system [2, 7, 8]. Histologically, they can be cystic, capillary, or cavernous [7]. Retroperitoneal lymphangiomas are rare and are more commonly seen before the age of 20 years. Our case was a 49-year-old woman with retroperitoneal lymphangioma, presenting with abdominal pain. Our patient’s age, her positive family history for pancreatic ductal adenocarcinoma, and location of the tumor, which was adjacent to the head of the pancreas, raised suspicion of other cystic neoplastic tumors like intraductal papillary mucinous neoplasms (IPMNs), mucinous cystic neoplasms (MCNs), and pancreatic adenocarcinomas. Other benign lesions including pseudocysts, hydatid cysts, and cavernous hemangiomas were included in the differential diagnosis of the reported lesion since further findings, such as normal appearance of PD and CBD, abutment of celiac branches, and elastography strain ratio of about 22, were confusing and not truly indicative of malignant or benign lesions. The wide range of differential diagnoses for the detected lesion made us proceed with EUS-FNA, which is a more accurate diagnostic technique for evaluation of pancreatobiliary region. Unfortunately, samples obtained from FNA did not provide a definite diagnosis. Inconclusive results and significant symptoms of the patient, which were attributed to the lesion, necessitated the surgical resection of the tumor. Histologic evaluation eventually revealed the lymphatic origin of the lesion, including large lymphatic channels in loose connective tissue stroma with peripheral lymphoid aggregations, which were compatible with lymphangioma. A large number of cases with retroperitoneal lymphangioma are asymptomatic, so these tumors are incidentally discovered when imaging has been performed for other reasons. Among the rare reported symptoms, abdominal pain is more common and is attributed to the compressive effect of the mass to the adjacent organs. An acute abdomen would be anticipated following probable complications like intestinal obstruction, cystic infection, intracystic/intraperitoneal/retroperitoneal hemorrhage, torsion, and cystic rupture [4]. Per the revised literature, hematoma, abscesses, duplication cysts, ovarian cysts, teratoma, mesothelioma, cystic metastases, lymphangiosarcoma, and pancreatic cystic neoplasms are considered as differential diagnoses of retroperitoneal lymphangioma [7]. In the literature, US study of lymphangioma usually reveals well demarcated, unilocular, or multilocular cysts with scattered echoes [9]. CT images reveal well-defined homogeneous cysts with prominent walls and distinct septations. These images can accurately provide definite information on the structure and location of these tumors [10]. MRIs also help to visualize these cysts, as they have distinct characteristics in *T*_1_ and *T*_2_ images [11]. Recurrence after complete and incomplete resection of the lymphangioma has been reported to be about 7% and 50%, respectively. So, free margin of surgically resected tumor is the cornerstone of the treatment [12]. One-year follow-up of the patient revealed complete resolution of the abdominal pain and no diagnostic features of recurrence on CT imaging.

## Conclusion

Retroperitoneal lymphangiomas are rare lesions with nonspecific symptoms and indistinct imaging clues, which make the diagnosis challenging. When located adjacent to the pancreas or peripancreatic major vessels, differentiation of pancreatic adenocarcinoma or pancreatic cystic lesions, which harbor a malignant potential, is of clinical importance. Histologic evaluation of the lesion maybe the gold standard of definite diagnosis. Surgical removal of the lesion is the standard method of therapy for symptomatic lesions.

## Data Availability

All the laboratory tests, imaging reports, and images are available in the archives of the patients referred to the Research Institute for Gastroenterology and Liver Diseases (RIGLD).
